# The Relation between Psychiatric Diagnoses and Constipation in Hospitalized Patients: A Cross-Sectional Study

**DOI:** 10.1155/2016/2459693

**Published:** 2016-03-10

**Authors:** Janique G. Jessurun, Peter N. van Harten, Toine C. G. Egberts, Ysbrand J. Pijl, Ingeborg Wilting, Diederik E. Tenback

**Affiliations:** ^1^GGz Centraal Psychiatric Center, Innova, 3818 EW Amersfoort, Netherlands; ^2^Division of Pharmacoepidemiology & Clinical Pharmacology, Utrecht Institute for Pharmaceutical Sciences, Faculty of Science, Utrecht University, 3584 CG Utrecht, Netherlands; ^3^Department of Psychiatry and Neuropsychology, Maastricht University, 6211 LK Maastricht, Netherlands; ^4^Department of Clinical Pharmacy, University Medical Center Utrecht, 3584 CX Utrecht, Netherlands

## Abstract

*Objective*. Constipation is a prevalent problem in patients with psychiatric disorders; it reduces quality of life and may lead to severe complications. The prevalence distribution of constipation across all psychiatric diagnoses in patients with severe mental illness (SMI) has hardly been studied. The aim of this study is to estimate the association between psychiatric disorders and constipation in SMI inpatients.* Methods*. The strength of the association between constipation (based on use of laxatives) and DSM-IV psychiatric diagnosis was studied in a cross-sectional study with “adjustment disorders” as the reference group. The association was analyzed using logistic regression.* Results*. Of the 4728 patients, 20.3% had constipation. In the stratum of patients older than 60 years, all psychiatric categories except for substance related disorders were significantly associated with a higher prevalence of constipation (odds ratios ranging from 3.38 to 6.52), whereas no significant associations were found in the stratum of patients between 18 and 60 years (odds ratios ranging from 1.00 to 2.03).* Conclusion*. In the elderly, all measured psychiatric diagnoses are strongly associated with an increased prevalence of constipation. Physicians should be extra alert for constipation in SMI patients, independent of specific psychiatric diagnoses.

## 1. Introduction

Constipation is a prevalent symptom in the psychiatric population, with a reported two-year period prevalence of 36.3% in schizophrenic patients [1] and 57.7% in depressive patients [2]. In addition, an incidence rate of 15 per 100 person-years has been observed in patients with severe mental illness (SMI) [3]. Although constipation is a frequent problem in the psychiatric population, there is little attention for it in research. This is in contrast with the clinical consequences of constipation that can cause or aggravate many common digestive symptoms and complaints and may even lead to severe complications such as fecal impaction, bowel perforation, paralytic ileus, and death [4] and is associated with a decreased quality of life in a similar degree as depression and diabetes [5]. In addition, SMI patients usually face delay in diagnosis and treatment of somatic comorbidity [6, 7]. A recent study has shown that only 18.5% of constipated psychiatric inpatients reported the presence of constipation to their psychiatrists [8]. The burden of the disease makes early recognition and treatment of constipation of clinical importance.

Dementia and depression are considered possible secondary causes of constipation [9]. In addition, anxiety and depression have been widely associated with functional gastrointestinal disorders, especially irritable bowel syndrome [10–12]. When diagnosing depression, constipation is usually taken into consideration, however, not with other psychiatric diagnoses. Despite the high incidence of constipation in psychiatric patients and the high burden of the disease, the distribution across all psychiatric diagnoses in the severe mentally ill has hardly been studied. The aim of this study is to assess the association between (primary) psychiatric diagnoses and constipation in psychiatric inpatients.

## 2. Materials and Methods

A cross-sectional study was performed at GGz Centraal “Zon en Schild,” a psychiatric center located in the Netherlands. Patients of 18 years and older hospitalized during 2007 and 2010, for whom a psychiatric diagnosis was available, were included. When patients were admitted multiple times during this observation period, data of the longest admission were taken into account. All data were obtained from the Electronic Healthcare Information System and from the Pharmacotherapeutical Response and Inventory Management system. The medical staff, according to DSM-IV criteria, entered diagnoses. Laxative use was taken as a proxy for constipation. Patients receiving at least one prescription for a laxative were considered having constipation.

The following potential confounders were evaluated [9]: gender, age, prescriptions for antidepressants, mood stabilizers (lithium, carbamazepine, lamotrigine, and valproic acid), benzodiazepines, antipsychotics, antispasmodics, motility inhibitors, anti-Parkinson drugs, hypnotics, antiepileptics, sympathicomimetics, antidiarrheal drugs, diuretics, calcium channel blockers, calcium supplements, iron supplements, antihistamines, opioids, nonsteroidal anti-inflammatory drugs, and comorbid somatic disorders including diabetes and hypothyroidism. Logistic regression analyses were used to evaluate the strength of the association between the psychiatric diagnoses and constipation. The patients being diagnosed with “adjustment disorders” were taken as the reference group; that is, the risk was set at 1, because (i) this kind of mental disorder is usually of short duration and a result of a specific stressor [13] and (ii) the disorder is relatively mild compared to other mental disorders [14]. Each covariate that univariately changed the odds ratios of the psychiatric diagnoses with more than 10% was incorporated into the final multivariate model. In addition, all analyses were checked for multicollinearity and interaction tests were performed across different age categories (18 to 60 years and 60 years or older). All data analyses were performed using SPSS version 18. This study was not subject to medical ethical review since (i) no data were collected in the course of a study, (ii) the patients did not have to change their behavior in any way, and (iii) all data were anonymous.

## 3. Results

A total of 5027 psychiatric patients were eligible. For 299 patients, no psychiatric diagnosis was available. Therefore, the final study population consisted of 4728 patients of which 959 patients (20.3%) had constipation. The results of the logistic regression analyses are presented in Table 1. Analysis of the categories “additional codes,” “other disorders in childhood,” “substance related disorders,” and “attention deficit disorders and disruptive behavior disorders” did not render representative results because of small numbers (less than 10 events). The interaction test between age and psychiatric diagnoses was significant (*p* < 0.05). Therefore, a stratified analysis was performed. For patients older than 60 years, all psychiatric diagnoses were significantly associated with an increased risk for constipation with odds ratios ranging from 3.38 to 6.52. However, in patients aged between 18 and 60 years, psychiatric categories were not significantly associated with an increased risk of constipation with odds ratios ranging from 1.00 to 2.03.

## 4. Discussion

This study shows that, in the elderly, all measured psychiatric diagnoses are significantly associated with constipation in comparison with patients with adjustment disorder. Among the young and middle-aged SMI patients, the strength of associations was much less and not statistically significant. The results of this first study on the association between psychiatric diagnosis and constipation are in contrast with clinical handbooks and previous studies that irritable bowel syndrome/constipation is more or less specifically related to depression and anxiety disorders [10–12]. Clinical handbooks do not report about the relationship between other psychiatric diagnoses and constipation [15].

Psychiatric disorders may be associated with constipation due to an effect of the disease itself and/or due to other associated factors. Patients with SMI generally have an unhealthy lifestyle (characterized by a poor diet, poor fluid intake, and inactivity) [16]. This may contribute to the occurrence of constipation [9]. No studies have been performed in psychiatric inpatients comparing the degrees to which these unhealthy lifestyle factors occur amongst the different psychiatric diagnoses and different ages. The results show statistically significant strong associations in the elderly but not in the young and middle aged. It can be hypothesized that these unhealthy lifestyle factors occur more frequently in elderly patients with psychiatric disorders [17], which may partially explain the results of this study. Another possible explanation for the results of the study is a differential use of psychotropic drugs in the elderly, as psychotropics are associated with the occurrence of constipation [3, 9]. Psychotropics with anticholinergic activities are widely believed to cause constipation to a different degree than psychotropics without anticholinergic activities; however, conflicting results have been reported [3]. Of the elderly patients and younger patients, respectively, 75.3% and 74.9% used psychotropics (i.e., antidepressants, antipsychotics, and/or mood stabilizers). The average duration of psychotropic drug use was much longer and the total defined daily doses were higher in those ageing 60 years or more. There is a possibility that physiological factors, such as colonic motility, anorectal physiology, cognitive impairment, and impaired rectal sensation, become more prevalent with increasing age. In addition, it is known that several comorbidities, which are associated with altered autonomic functioning (such as diabetes), become more prevalent with increasing age. It is also likely that the prevalence of past abdominal surgeries (which are associated with constipation) increases with age.

One limitation of this study is that there were no data regarding several potential risk factors like a high body mass index (BMI), poor diet, low physical activity, low socioeconomic status, and smoking status [9, 18]. However, all hospitalized patients receive a similar diet in this institution. Furthermore, diabetes, age, and psychotropic use (covariates in the analysis) are related to BMI and physical inactivity [19, 20].

A second limitation is the use of laxatives as a proxy for constipation. This has probably led to an underestimation of the incidence as SMI patients usually face delay in diagnosis and treatment of somatic comorbidity [6, 7]. Another possibility for underestimation is the use of over-the-counter laxatives. However, most inpatients have a low income and prefer to ask for a prescription for laxatives instead of buying laxatives themselves.

A third limitation may be the choice of the control group. However, the group of inpatients with adjustment disorders was chosen, because this group is far more comparable to the group of inpatients with other psychiatric diagnosis than healthy controls that are not admitted and do not have a psychiatric diagnosis. Average duration of hospitalization, duration of psychotropic drug use, and the average of total defined daily doses were much shorter/lower in the patients with adjustment disorders. The period prevalence of constipation was 6% in this group, which is lower than the reported prevalence of 15% in the Dutch general population [21]. These findings suggest a useful and valid reference group.

Finally, it is important to note the cross-sectional nature of the study, not enabling making conclusions regarding causation.

Although conclusions with regard to causation are limited, constipation is prevalent in patients with SMI and associated with a decreased quality of life. In addition, constipation may lead to severe complications and SMI patients usually face delay in diagnosis and treatment of somatic comorbidity [6, 7]. Thus, the burden of constipation makes early recognition and treatment of constipation of clinical importance. This study shows the importance of alertness on constipation in all elderly SMI patients. Possible solutions on reducing the incidence and complications of constipation could be creating more awareness and thereby earlier recognition of constipation and stimulating healthier lifestyle. Treatment options of constipation include lifestyle modifications, the use of laxatives, and cessation/dose-reduction of contributing medication [9].

## 5. Conclusion

In elderly psychiatric inpatients, all measured psychiatric diagnoses are significantly associated with an increased prevalence of constipation. Physicians should be extra alert for constipation in this population. This alertness should not be limited to specific diagnostic groups or specific psychotropics.

## Figures and Tables

**Table 1:** The association between psychiatric diagnoses and constipation (stratified for age).

Psychiatric diagnoses	18 to 60 years		60 years and older	
	(*n* = 3433)		(*n* = 1295)	
	Period prevalence of constipation (% (*n*/*N*))	Adjusted odds ratio (95% confidence interval)^a^	Period prevalence of constipation (% (*n*/*N*))	Adjusted odds ratio (95% confidence interval)^a^
Adjustment disorders	6.0% (16/267)	1.00 (reference)	5.7% (5/87)	1.00 (reference)
Schizophrenia and other psychotic disorders	16.1% (170/1054)	1.52 (0.83 to 2.78)	49.8% (142/285)	5.72 (2.01 to 16.28)^*∗*^
Depressive disorders	12.5% (65/518)	1.28 (0.70 to 2.33)	40.9% (110/269)	4.94 (1.77 to 13.73)^*∗*^
Personality disorders	17.2% (94/548)	1.68 (0.92 to 3.08)	47.1% (32/68)	5.20 (1.68 to 16.12)^*∗*^
Delirium, dementia, and amnestic and cognitive disorders	13.2% (21/159)	1.42 (0.68 to 2.99)	34.6% (92/266)	4.45 (1.58 to 12.50)^*∗*^
Alcohol-related disorders	13.8% (29/210)	1.56 (0.78 to 3.13)	18.7% (29/155)	3.59 (1.21 to 10.68)^*∗*^
Bipolar disorders and other mood disorders	19.4% (48/247)	1.60 (0.91 to 3.15)	40.2% (35/87)	3.38 (1.08 to 10.57)^*∗*^
Anxiety disorders	10.7% (17/159)	1.14 (0.53 to 2.45)	32.7% (17/52)	4.18 (1.28 to 13.66)^*∗*^
Pervasive developmental disorders	12.8% (12/94)	2.03 (0.84 to 4.90)	100% (1/1)	NE
Rest group	9.7% (7/72)	1.00 (0.36 to 2.82)	45.0% (9/20)	6.52 (1.58 to 27.00)^*∗*^

^a^All categories were adjusted for gender, age, the use of antipsychotics, mood stabilizers, antidepressants, calcium supplements, iron supplements, diuretics, NSAIDs, opioids, lithium, sympathicomimetics, anticholinergics, and benzodiazepines.

^*∗*^Significant at a 5% level.

NE = not estimable.
